# Prescribing benzodiazepines in young adults with anxiety: a qualitative study of GP perspectives

**DOI:** 10.3399/BJGP.2024.0211

**Published:** 2024-10-01

**Authors:** Charlotte Archer, Nicola Wiles, David Kessler, Carolyn A Chew-Graham, Katrina Turner

**Affiliations:** Centre for Academic Mental Health, University of Bristol, Bristol Medical School, Bristol.; Centre for Academic Mental Health, University of Bristol, Bristol Medical School, Bristol.; Centre for Academic Mental Health, University of Bristol, Bristol Medical School, Bristol.; School of Medicine, Keele University, Staffordshire.; Centre for Academic Primary Care, University of Bristol, Bristol Medical School, Bristol, and National Institute for Health Research, Applied Research Collaboration West, University Hospitals Bristol NHS Foundation Trust, Bristol.

**Keywords:** anxiety disorders, benzodiazepines, general practice, prescribing, primary health care, qualitative research

## Abstract

**Background:**

Incident benzodiazepine prescriptions in primary care for anxiety decreased between 2003 and 2018. However, from 2008, incident prescribing of benzodiazepines for anxiety increased among those aged 18–34 years. There are increasing concerns around prescribing of benzodiazepines. Further, although guidelines state benzodiazepines should only be prescribed short term, in 2017, 44% of incident prescriptions were prescribed for longer than the recommended duration of 2–4 weeks.

**Aim:**

To understand when and why GPs prescribe benzodiazepines for anxiety in young adults.

**Design and setting:**

A qualitative study was undertaken using in-depth interviews with 17 GPs from 10 general practices in South West England.

**Method:**

Interviews were conducted by telephone or videocall. A topic guide was used to ensure consistency across interviews. Interviews were audio-recorded, transcribed verbatim, and data analysed using reflexive thematic analysis.

**Results:**

GPs described caution in prescribing benzodiazepines for anxiety in young adults, but thought they had an important role in acute situations. GPs described caution in prescribing duration, but some thought longer-term prescriptions could be appropriate. In light of these views, some GPs questioned whether primary care needs to revisit how clinicians are using benzodiazepines. GPs perceived that some young adults requested benzodiazepines and suggested this might be because they wanted quick symptom relief. GPs noted that refusing to prescribe felt uncomfortable and that the number of young adults presenting to general practice, already dependent on benzodiazepines, had increased.

**Conclusion:**

Patient-driven factors for prescribing benzodiazepines suggest there are current unmet treatment needs among young adults with anxiety. Given increases in prescribing in this age group, it may be timely to revisit the role of benzodiazepines in the management of people with anxiety in primary care.

## Introduction

The prevalence of anxiety is high (UK prevalence is 7.2%)^1^ and is typically managed in primary care.^2^ The National Institute for Health and Care Excellence (NICE) guideline for anxiety recommends that first-line treatment offered for anxiety should be a psychological intervention.^3^ Medication is a second-line option, whereby selective serotonin reuptake inhibitor antidepressants (SSRIs) are usually prescribed.^3^ Other NICE-recommended drugs for anxiety include serotonin and norepinephrine reuptake inhibitors (SNRIs), pregabalin, or benzodiazepines such as diazepam.^4^^,^^5^ However, NICE and *British National Formulary* (*BNF*) guidelines state that benzodiazepines should only be prescribed short term, that is, 2–4 weeks, owing to risks of dependency and toxicity.^3^^,^^6^ Benzodiazepines can also cause severe side effects, such as sedation and memory problems,^7^ and patients may struggle with the withdrawal of treatment, potentially experiencing rebound anxiety.^8^ There is also evidence that benzodiazepine use is associated with other adverse health effects, including increased acute care or psychiatric admissions, polypharmacy, and poorer quality of life.^9^^,^^10^ This increase in adverse events has been reported in both older and younger adults.^11^

Historically, benzodiazepines were often prescribed for anxiety.^12^ However, owing to increasing concerns around their potential for abuse, there has been a move away from using this drug in general practice. GPs in the UK report being cautious of initiating benzodiazepines, being vigilant in their monitoring when they do initiate treatment, and do not consider long-term prescribing appropriate.^13^ Indeed, using anonymised patient data from UK general practice records, between 2003 and 2018, incident benzodiazepine prescriptions for anxiety in adults of all ages decreased from 6.4 per 1000 person–years at risk (PYAR) to 4.6 per 1000 PYAR.^14^ Further, the proportion of short-term benzodiazepine prescriptions increased over the 16 years, while longer-term prescribing decreased,^14^ again, in adults of all ages. That said, in 2017, 44% of incident prescriptions for benzodiazepines were still prescribed for longer than the recommended 4-week maximum, and more than 20% were prescribed for longer than 6 months.^14^

**Table:** How this fits in

Between 2003 and 2018, overall incident benzodiazepine prescriptions for anxiety decreased, yet prescribing in young adults has increased since 2008. Further, nearly half of all new prescriptions in 2017 were prescribed for longer than the recommended duration. Our study shows that, while GPs express caution in their prescribing of benzodiazepines, they view them as having a role in helping young adults manage acute anxiety symptoms, and that they may be helpful on a longer-term basis. Our research identified perceived patient-driven reasons for prescribing benzodiazepines, and these suggest young adults with anxiety in UK primary care may have unmet management and support needs.

Given the associated harms, it is concerning that prescribing of benzodiazepines for anxiety in young adults is at odds with the overall decline seen. New prescriptions for those aged 18–24 years increased from 3.2 per 1000 PYAR in 2008 to 4.6 per 1000 PYAR in 2018, and from 4.2 per 1000 PYAR to 6.2 per 1000 PYAR in those aged 25–34 years during the same time period.^14^ The number of young adults diagnosed with anxiety and depression has more than doubled over the past decade,^15^^,^^16^ but the reasons behind the increase in incident benzodiazepine prescribing are not known. Although previous qualitative studies have explored practitioners’ reasons for prescribing benzodiazepines, particularly in older adults,^17^^,^^18^ we do not know why GPs may choose to prescribe a benzodiazepine for anxiety in younger adults. The aim of this study was to explore GPs’ views on when and why they prescribe these drugs to young adults, aged 18–34 years, with anxiety.

## Method

### Recruitment and sampling

Around 30 general practices located in South West England were informed about the study by the local Clinical Research Network (CRN) and Bristol, North Somerset and South Gloucestershire’s Integrated Care Board (ICB). GPs working in these practices, who were willing to be interviewed, emailed or telephoned the research team. Purposive sampling^19^ took place. Those selected for interview were emailed a study invitation and information sheet. A date and time for the interview to take place were arranged shortly after. GPs were given £40 to reimburse them for the time taken to participate.

While data collection was ongoing, the lead researcher asked the CRN and ICB to promote the study to GPs who were under-represented, in relation to who had already been interviewed. The aim was to ensure the final sample included interviewees who varied in terms of gender, age, number of years practising, and in relation to their practice deprivation score, list size, and demographic characteristics of their patient populations.

### Data collection

The interviews were conducted by the lead researcher (a mixed-methods researcher experienced in interviewing practitioners), and took place either over the telephone or by videocall. The researcher introduced the project aims in the introductory section of the interview and told interviewees that she was not a medic or an expert in the prescribing of psychotropic medication. A topic guide was used to ensure consistency across the interviews. The guide was developed from the aims of the study. It was also developed from discussions within the research team and with the patient and public involvement (PPI) group (*n* = 4 with lived experience of anxiety) (Supplementary Box S1). It included questions about what factors influence GPs’ prescribing decisions, the risks and benefits of prescribing benzodiazepines for longer periods, and when they are used among all patients, but particularly young adults. Interviewees were asked to complete a brief demographic questionnaire after the interview. The information collected was used to describe those interviewed and to purposively sample further interviewees.

### Data analysis

Drawing on the principles of grounded theory, data collection and analysis proceeded in parallel so that insights from early interviews could inform the focus of later data collection.^20^ Doing this also meant data collection ended when data saturation was reached, where no new themes were identified in later interviews. All interviews were audio-recorded using an encrypted voice recorder, transcribed verbatim, fully anonymised, checked for accuracy, and analysed using reflexive thematic analysis, as defined by Braun and Clarke.^21^

The lead author and another author (a senior qualitative methodologist) independently read and re-read a sample of transcripts to familiarise themselves with the data, and using an inductive data-driven approach they manually noted possible codes. They then met to compare and discuss their coding and interpretation of the data. Any differences in coding were resolved through discussion, the addition of further codes, and clarification of existing ones. A preliminary coding framework was developed and discussed, and this was later revised slightly, as new codes were identified in subsequent transcripts. Transcripts that had previously been coded were re-coded with the updated framework where necessary.

NVivo (version 12) was used to electronically code transcripts so that quotes relating to each code could be easily extracted. The extracted data were then organised within a table where each row represented individual interviewees and columns represented specific codes. This is an approach to organise data based on framework analysis,^22^ although analysis was still undertaken using reflexive thematic analysis.^21^ Data in the table were read and re-read to identify themes between and within interviewees, and to identify deviant cases. Where there was a spectrum of views within the main themes, they are reported as the views of ‘a few GPs’ rather than ‘many’ or ‘most’. These themes and extracts of the data were discussed with the wider research team and the PPI group, with themes revised and refined as a result of these discussions. The PPI contributors felt that the results were important and relevant. Reflexive notes were kept during data collection, analysis, and interpretation of data to enable decisions to be audited throughout the process and to enhance the rigour and trustworthiness of the research.^23^

## Results

In total, 17 GPs from 10 general practices were interviewed between August 2022 and February 2023 (Table 1). Three of the GPs worked in a practice that had a large university student population. Eleven GPs interviewed were female, and interviewees were aged between 32 and 60 years (mean age: 46.7 years, standard deviation (SD): 8.9 years). Just over half of those interviewed were salaried GPs, with one describing themselves as a *‘long-term locum’* and one reporting having an additional qualification in mental health. Interviewees had been working as GPs for between 6 months and 30 years. Around half of GPs (*n* = 9) had a practice deprivation score of ≤5, which meant they were situated in more deprived areas.^24^^,^^25^ The mean length of the interviews was 30 minutes.

**Table 1.** Characteristics of GPs interviewed and practice deprivation deciles

**Characteristic**	***n***
**Sex**	
Female	11
Male	6

**Age, years**	
30–39	6
40–45	4
50–59	6
≥60	1

**Ethnicity (self-disclosed)**	
White	14
Asian	2
Mixed	1

**Role in practice**	
Partner	8
Salaried GP	9

**Practice deprivation score**	
1–3	5
4–7	4
8–10	8

a *Deprivation score for the practice patient population where 1 indicates the most deprived patient population and 10 the least deprived. Taken from the* National General Practice Profiles,*24 which calculated scores based on the 2015 English Indices of Deprivation.^25^*

Findings from the interviews are presented under two headings, GP-driven prescribing and patient-driven prescribing, with three subheadings under each (Box 1). These subheadings reflect themes identified in the data that were factors in GPs deciding to prescribe benzodiazepines for patients with anxiety, particularly in young adults.

**Box 1.** The six themes identified in the interviews relating to GP prescribing of benzodiazepines

**GP-driven prescribing**	**Patient-driven prescribing**
A role to play in acute anxiety	Young adults want benzodiazepines
Managing risk with prescribing duration	Patient pressure to prescribe
Revisiting the use of benzodiazepines	Prior dependence on benzodiazepines

### GP-driven prescribing

#### A role to play in acute anxiety

Many GPs stated that they tried not to use benzodiazepines for anxiety. Most GPs, however, noted that there were times when they felt a short-term benzodiazepine prescription was indicated, and that the benefits would outweigh the risks. These situations were ones where the individual was experiencing acute anxiety symptoms (such as following a bereavement), when they were starting SSRI treatment, or in a crisis situation where the individual was struggling to cope and unable to immediately access mental health services:

*‘In crisis times … you’re already not coping, not sleeping, perhaps this would kick you back into a bit more of a sleep rhythm, we all know that sleep deprivation is a form of torture therefore you just need a couple of days sleep … here* [is some] *diazepam whilst I refer you on to PCLS* [primary care liaison service].*’*

(GP 12, female, 7 years practising [YP])

#### Managing risk with prescribing duration

When talking about the length of these short-term prescriptions, most GPs referred to *‘three days or something just to take the edge off it’* (GP 6, female, 20 YP) or up to ‘*five days’ worth*’ (GP 1, female, 18 YP). Some GPs suggested they might be inclined to prescribe benzodiazepines in young adults on a more medium-term basis, if they thought it would help them get through a particularly difficult period:

*‘They might be on them for weeks … supposing there was a man whose relationship had broken down … lost their job … sleeping in their car because they have got nowhere else to live …* [it is a] *pretty awful situation. They are trying to get back on their feet and benzos is one thing that does help them. And in a way it is better that than drinking.’*

(GP 13, male, 28 YP)

Some GPs also noted that longer-term prescribing, sometimes for many months, was also taking place in young adults; they suggested that this was owing to a lack of alternative treatment available, in either primary or secondary care:

*‘I also have a number of patients … frequent attenders … people that are very difficult to be helped because they have now got resistant anxiety and lots of issues that the therapies simply don’t exist for … you have to be really quite severe* [to] *meet secondary care criteria … they are a massive cohort that fall through the gap and those are the people that are actually quite resistant to all of the other methods and they need lots of benzodiazepines continuously.’*

(GP 8, female, 14 YP)

Nonetheless, GPs were, on the whole, against long-term prescribing. Some GPs explained that their practices had explicit statements about not being willing to prescribe benzodiazepines long term, and that, if patients registered with them who were already on a long-term prescription, then they would actively work with them to reduce that:

*‘We have a letter in our notes … “we are not a surgery that prescribes long-term benzos, we need to work with you in partnership to reduce your benzodiazepines”… we would try and reduce by ten per cent per month … we do not do it all the time, we could do it more … I do have some patients who are on long-term benzos and I have given up trying that because you try it and they get worse … it is difficult.’*

(GP 9, female, 13 YP)

When patients were on a long-term benzodiazepine prescription, GPs said that it was usually the practice’s *‘clinical pharmacist* [who] *will speak to them about starting a reduction programme’* (GP 12, female, 7 YP). GPs suggested that their pharmacy teams were usually more successful than the GPs at getting patients to reduce and come off the drug, although the patient still needed to be willing:

*‘Clinical pharmacists seem to get them off it better … it is like seeing a specialist … “if the pharmacology person says this, maybe should I come off or wean down my benzo”, but if your GP says that, it causes a lot of hassle and work … but … unless the patient is willing to work with you on it is very difficult*.*’*

(GP 6, female, 20 YP)

#### Revisiting the use of benzodiazepines

Some GPs questioned whether concerns in the medical community around benzodiazepines had gone *‘too far’* (GP 7, female, 28 YP). One GP noted comments made by an academic psychiatrist:

*‘I always remember him saying that “in a way benzos have got a bad name really, and that actually, they are quite an effective drug”. So … I do sometimes prescribe benzos for people on a more medium-term basis really.’*

(GP 13, male, 28 YP)

GPs commented that benzodiazepines have a place for managing the more acute and severe symptoms of anxiety, and that, despite the NICE guidelines, sometimes longer-term prescribing could be appropriate:

*‘We are getting more patients coming out on long-term benzodiazepines … they can be quite helpful in those situations … for a while I was really like, hands off … benzodiazepines, but actually now I am a bit more comfortable with them and … with patients actually not getting addicted to them … in the right circumstances and with the right counselling, they are a really effective medication.’*

(GP 17, female, 10 YP)

Most GPs acknowledged that secondary care seemed quite comfortable to prescribe benzodiazepines for anxiety and had been *‘prescribing benzodiazepines more, recently’* (GP 17, female, 10 YP):

*‘There are definitely patients who come from secondary care who have it as part of their management to be on it more regularly, which it feels like secondary care are happier to do that.’*

(GP 14, male, 6 YP)

Therefore, there was a sense that if secondary care is prescribing these drugs, then *‘have we* [primary care] *gone too far the other way, are we not using benzodiazepines, when actually they could perform a role and a function?’* (GP 7, female, 28 YP).

### Patient-driven prescribing

#### Young adults want benzodiazepines

GPs reported an increase in the number of young adults presenting to primary care with anxiety, with *‘lots of young adults seeking benzodiazepines’* (GP 11, male, 0.5 YP). There was a sense that there had been a shift in how the younger generation wanted their anxiety managed, compared with previous generations. They explained that this age group would often *‘demand’* them *‘because they know they work, and a patient beautifully put it to me* [the GP]*, “I want something that works today, I want something that works quickly, and I want something that just takes this away for me.” And that is the society we live in now’* (GP 8 female, 14 YP).

GPs explained that, when patients requested benzodiazepines, they tried to address the underlying cause of why they were asking for them, rather than just *‘putting a sticking plaster over the issue’* (GP 16, male, 5 YP). GPs said they would often suggest talking therapies or SSRIs as an alternative to benzodiazepines. However, GPs reported that, when explaining to young people that there were substantial waits for talking therapies, and that there was a delay in SSRIs taking effect, younger people were put off by these other options:

‘*We often talk to patients about the fact that they* [antidepressants] *are a long-term prescription and that there is delayed onset of action …* [there is a] *generation of people wanting things fixed quickly and therefore a benzodiazepine is likely to give them some short-term relief … that’s why they are being used in younger adults*.’

(GP 4, male, 25 YP)

That said, GPs also noted that some young adults were aware of the risks of starting benzodiazepines. They understood that they could be potentially addictive and did not always want to try them:

*‘This group of young people are really receptive to the idea that you can be drug dependent and that starting them in your teens, twenties, thirties without knowing that it can lead to dependence, can be really harmful.’*

(GP 2, female, 1 YP)

#### Patient pressure to prescribe

Most GPs reflected that it would be very difficult to manage a consultation where a young adult really wanted a benzodiazepine prescription. They said that *‘the vast majority of requests for benzos in general come from the patient’* (GP 7, female, 28 YP) and often patients would say *‘in the past Dr So and So has given me diazepam … I will look at their records and, sure enough, Dr So and So has done that’* (GP 15, male, 27 YP). GPs explained that this put them in a position where they had to *‘fight’* to not prescribe, and that *‘sometimes it’s very difficult to win that fight … people will go to great lengths to get this drug from you’* (GP 8, female, 14 YP).

Many GPs described how young adults would consult with different GPs each time, to try to get another benzodiazepine prescription for their anxiety. This meant that patients were actually being prescribed them *‘much more than we think we’re giving them’* (GP 3, female, 30 YP):

*‘The trouble is they often re-present and get some more from somebody else … you suddenly find they have had had five lots and everybody has gone “no this is the last one, you cannot have anymore”… it often feels like a battle … an uncomfortable consultation when they’re asking for them.’* (GP 3, female, 30 YP)

When GPs reported that they declined to prescribe benzodiazepines, those patients could become the *‘difficult ones’* (GP 5, female, 20 YP) whereby they might refuse to leave the consultation room or become angry. There was a sense from several GPs that there was a particular skill required for GPs to be able to handle this situation without prescribing a benzodiazepine. The GP with an additional qualification in mental health explained that:

*‘It is not because they want to benzo prescribe, but from what I’ve noticed, it is colleagues finding it difficult to deal with this. When somebody is in that acute stress reaction in front of you, not many doctors have the skill of talking them down … there needs to be an upskilling in training for doctors in order to deal with mental health, rather than prescribe for mental health.’*

(GP 8, female, 14 YP)

#### Prior dependence on benzodiazepines

GPs working in practices located in more deprived areas talked about the increasing number of young adults that were self-medicating from *‘street’* or internet-acquired benzodiazepines. By the time they presented to the GP, they were already dependent on them:

*‘They are very freely available … in the young population … they are using Xanax, there is more and more of those we are finding. Those that have got benzodiazepine dependence because they have been using things they have bought and then they are trying to come off.’*

(GP 5, female, 20 YP)

These GPs said this was a *‘big problem’* (GP 11, male, 0.5 YP) for their practices, and had led to them being stricter with their initial prescribing of benzodiazepines, as they did not want to be *‘contributing towards our community’s problem with that’* (GP 11, male, 0.5 YP). They explained they tried to work with these young adults to help reduce their dose:

*‘For our inner-city practice population, the commonest benzo prescriptions are for people who are buying them on the street and then we agree to do a planned reduction.’*

(GP 7, female, 28 YP)

GPs were aware that, if they did prescribe benzodiazepines, then they could get a reputation as a GP who prescribed this drug:

*‘With our patient population, if you start prescribing diazepam, word gets round that if you go to Dr X, then you will get given benzos.’*

(GP 10, female, 25 YP)

GPs also said they were worried that there was too much street value in benzodiazepines, and when patients said things like *‘only the five milligrams work for me, not the two milligrams, even if I take the equivalent dose in two milligrams* [tablets]*’* (GP 12, female, 7 YP), GPs were concerned that patients wanted to sell them. A few GPs explained that their practices had prescribing policies to try to manage this situation:

*‘If they do ask* [for benzodiazepines] *I always wonder if they have an ulterior motive … getting them to sell … abuse them … get high … we have a practice policy in that we only give out the two milligram tablets, so even if someone needs five milligrams they get prescribed two-and-a-half two milligram tablets, because they have less street value.’*

(GP 11, male, 0.5 YP)

## Discussion

### Summary

GPs describe caution in prescribing benzodiazepines to young adults with anxiety and a reluctance to prescribe benzodiazepines on a longer-term basis. However, in the context of long waiting lists to access talking therapies, and perceived limited treatment options for individuals in acute distress, GPs think benzodiazepines have an important role in the management of anxiety in young adults. GPs describe giving short-term prescriptions to reduce the risk of dependency, but some GPs suggest that medium- or longer-term prescribing can be appropriate when the benefits are perceived to outweigh the risks. In light of these views, GPs question whether primary care needs to revisit how clinicians are using benzodiazepines, particularly when the drug has been initiated in secondary care.

GPs report that many young adults present to general practice wanting a benzodiazepine prescription for anxiety. GPs suggest this is because younger people want their symptoms to be resolved more quickly than previous generations, and do not want to wait to access NHS talking therapies or for an antidepressant to take effect. GPs note it can feel uncomfortable to refuse to prescribe a benzodiazepine, particularly if the patient is persistent. GPs reflect on the recent increase in the number of young adults presenting to primary care who are already dependent on benzodiazepines, having acquired them outside primary care.

### Strengths and limitations

The semi-structured nature of the interviews enabled participants to raise issues that were important to them, and to describe their views in detail. The GPs interviewed varied in terms of their age, number of years practising, roles, and deprivation decile of their practice. The variation on the latter variable was important, given the additional factors influencing GPs’ prescribing decisions in more deprived areas. Data collection and analysis took place in parallel, and continued until data saturation had been reached. However, only one-third of the interview participants were male. In addition, those interviewed were volunteers, so may have been GPs with a special interest in this area, or GPs who were concerned about when and how often benzodiazepines are prescribed.

### Comparison with existing literature

Previous qualitative research, which was undertaken more than 10 years ago with UK GPs around prescribing benzodiazepines, reported that GPs were cautious in initiating these drugs, they were vigilant in their monitoring when they did prescribe, and did not consider long-term prescribing appropriate.^13^ The GPs in our study reported similar views on prescribing benzodiazepines for anxiety and, on the whole, were also reluctant to prescribe on a long-term basis. Previous research has argued that the risk-to-benefit ratio for benzodiazepines means they are not appropriate for most clinical situations, and suggested that they should become a controlled substance to reduce potentially *‘dangerous prescribing’*.^26^ Although the GPs in our study did not express this view, they noted the risk of dependency.

However, some GPs suggested that primary care needs to revisit how benzodiazepines are being used for anxiety, with the potential for them to be used more than they currently are. This is in line with the stance that others have adopted, with Starcevic^27^ proposing that the NICE anxiety guidelines could be reviewed to include benzodiazepines as a first-line, long-term treatment, given the evidence for their efficacy when used as a combination therapy, and the lack of evidence for other anxiolytics’ superiority in treating anxiety. That said, the views on revisiting benzodiazepine use shared by some of the GPs in our study were discussed in the context of increasing prescriptions initiated by secondary care. Studies have highlighted concerns between the primary and secondary care prescribing interface, whereby GPs are expected to prescribe higher-risk medication based on secondary care advice, with neither the GP nor the specialist feeling responsible for deprescribing of such medication.^28^^,^^29^ Reducing benzodiazepine prescribing has been identified as a key medication optimisation opportunity for the NHS in 2023–2024^30^ and GPs in our study noted that practice pharmacists could have more success in working with patients to reduce their benzodiazepine use. This supports findings from the existing literature that pharmacists can play a key role in reducing prescribing of this drug and can improve patient outcomes.^31^^,^^32^

There has been an increase in the number of young adults diagnosed with anxiety in recent years^15^ and this may, in part, explain the increase in young adults being prescribed benzodiazepines,^14^ particularly if their symptoms are severe or acute. GPs in our study talked about an increase in the numbers of younger people presenting to primary care with anxiety, and that this age group specifically requested benzodiazepines, as they want something that would work quickly and did not want to wait for talking therapy or SSRIs to take effect. These findings are in line with a Belgian study, which reported that GPs were reluctant to prescribe benzodiazepines, but did so because of the lack of alternatives that would work quickly, and insufficient time to spend with their patients to address psychosocial problems.^18^

In our study, GPs reflected on the discomfort they experienced when refusing a patient’s request, such as declining to prescribe a benzodiazepine. Findings from a qualitative research synthesis to understand GPs’ experiences of prescribing this drug (in patients of all ages and conditions) also reports GPs feeling ‘uncomfortable’ when they want to help patients but felt responsible for minimising benzodiazepine use.^33^ In addition, an intervention has been developed to train GPs to handle mental health consultations without prescribing a benzodiazepine, and offering psychological interventions instead,^34^ indicating that others have recognised the challenges GPs may experience when refusing to provide a benzodiazepine prescription. The fact that GPs in our study talked about young adults presenting to general practice already dependent on benzodiazepines, having acquired them from the ‘*street*’, suggests refusing a prescription might mean patients seek alternative illegal sources. Benzodiazepine misuse is most common in young adults,^35^ and increasing numbers of young adults are experimenting with benzodiazepines in the UK,^36^ the Republic of Ireland,^37^ and the US.^38^

### Implications for research and practice

Although GPs say they are cautious in initiating benzodiazepines and reluctant to prescribe them for longer than the recommended 2–4 weeks, the rate of prescribing in young adults is increasing, and almost half the prescriptions are for longer than 4 weeks. There are a number of possible contributory factors discussed by GPs in this study, including the difficulty of accessing talking therapies because of waiting times, the rapid effect of these drugs in difficult situations, and pressure from patients. Nonetheless, the potential for creating long-term dependency in young adults is worrying and it is important that, when GPs initiate a benzodiazepine prescription, they continue to monitor the risks and benefits for ongoing use and prescribe the smallest dose and shortest duration for clinical benefit.

Given the increase in prescribing, and the reflections from GPs on the increasing use of these drugs in secondary care, it may be timely to reconsider when and how benzodiazepines should be used in primary care for the management of people with anxiety, particularly young adults. Research with young adults to understand their views would be key to informing any changes in guidelines. It may also indicate a current unmet need for the support and management of young adults with anxiety in UK primary care and commissioners should consider how existing services can be adapted to better support this age group.

## Data

Anonymised qualitative interview transcript data will be stored on the University of Bristol’s Research Data Service repository. Bonafide researchers will be able to access this data subject to a data access agreement and following approval from the University of Bristol Data Access Committee.
